# Amino acid impact factor

**DOI:** 10.1371/journal.pone.0198645

**Published:** 2018-06-13

**Authors:** C. K. Sruthi, Meher Prakash

**Affiliations:** Theoretical Sciences Unit, Jawaharlal Nehru Centre for Advanced Scientific Research, Bangalore-560064, India; UMR-S1134, INSERM, Université Paris Diderot, INTS, FRANCE

## Abstract

Amino acid mutations in proteins are random and those mutations which are beneficial or neutral survive during the course of evolution. Conservation or co-evolution analyses are performed on the multiple sequence alignment of homologous proteins to understand how important different amino acids or groups of them are. However, these traditional analyses do not explore the directed influence of amino acid mutations, such as compensatory effects. In this work we develop a method to capture the directed evolutionary impact of one amino acid on all other amino acids, and provide a visual network representation for it. The method developed for these directed networks of inter- and intra-protein evolutionary interactions can also be used for noting the differences in amino acid evolution between the control and experimental groups. The analysis is illustrated with a few examples, where the method identifies several directed interactions of functionally critical amino acids. The impact of an amino acid is quantified as the number of amino acids that are influenced as a consequence of its mutation, and it is intended to summarize the compensatory mutations in large evolutionary sequence data sets as well as to rationally identify targets for mutagenesis when their functional significance can not be assessed using structure or conservation.

## Introduction

Anfinsen’s dogma of molecular biology postulates that the native structure and function of proteins are uniquely determined by its amino acid sequence. [1] As such there is a lot of fundamental interest in analysing the sequences of proteins. For example, sequence data of protein from multiple species helps in understanding evolutionary patterns and that from a cohort helps with the drug resistance patterns. Multiple Sequence Alignment (MSA) of protein sequences obtained from across species or a cohort is usually the starting point for many such analyses. The simplest analysis one can perform using MSA is evaluating the level of conservation of the individual amino acids. A highly conserved amino acid is likely to have an important role either in structure or in function, and it is especially true for the perfectly conserved amino acids that are mostly identified in the functional sites of proteins. Based on similarity and homology of sequences curated from different species, protein sequences are classified into families which are likely to share structural and functional similarities. The interest in the functional information contained in the sequence analysis is only enhanced by the next generation sequencing technology [2] which is making sequence data easily accessible compared to the structural data.

Most sites tolerate a degree of change, which reflect either a polymorphism or a mutation under selection pressure. But co-evolution, where another amino acid simultaneoulsy undergoes a mutation along with it, is gaining a lot of recent interest. Statistical methods such as Statistical Coupling Analysis (SCA) [3] and Direct Coupling Analysis (DCA) [4] use large sequence sets of homologous sequences to uncover several biological details about proteins. SCA considers thousands of similar sequences in order to derive an energetic coupling between any two amino acids. Clusters of amino acids contributing specifically to structural stability or catalysis efficiency could be identified. [5] This led to the development of the concept of functionally coupled domains of proteins called Protein Sectors. [5] Direct Coupling Analysis (DCA) has been used specifically to identify the structural protein-protein contacts. In DCA the secondary correlations between noninteracting residues are removed to identify the residue pairs which are near in structure or which interact directly. Recent studies, some based on DCA, [6] have used pairwise co-evolution of different amino acids for *de novo* protein structure prediction, without using structural homology information. [6, 7] Alternative to these statistical methods are combinatorial methods, which had a general applicability from amino acid co-evolution [8] in proteins to species co-evolution. [9] The methods combined co-evolution information with phylogeny to circumvent the limitation on the number of sequences. [8] The combinatorial methods applied to proteins not only recovered the functional networks of amino acids predicted by SCA, but could identify other functionally related networks that were otherwise considered to be lost due to lack of sequence divergence. [8]

Several co-evolutionary analysis algorithms have been developed, [10] some methods combined the predictions from different algorithms to identify drug resistance patterns in a cohort. [11] Mutual information based methods have also been developed to identify covarying residues. [12, 13] Bayesian networks were used to construct models which show directional dependencies between amino acids, with potential implications for HIV-1 drug resistance. [14] While potentially Bayesian networks can be very powerful, the directional relations may not be robust nevertheless. [14] Furthermore, they solve inverse problem from the data which may not be as intuitive as the co-evolution networks.

Asymmetric or directional dependencies effects have been studied in other biological contexts such as in gene expression data [15] and regulatory networks. [16] However, in the context of mutations, all the efforts focused on developing symmetric co-evolution measures of amino acid pairs, and they can not suggest a directed relation between them. The idea is to capture how many amino acids *j* are likely to undergo a compensatory change in response to a change in amino acid *i*, which may be important for structure or function of the protein. These relations can be obtained between pairs of amino acids that are either within the same protein or between two interacting proteins. In order to visualize the (un)directed co-evolutionary relations, an effective tool is network representation, which has been used in metabolic interaction networks, [17] protein-protein interaction networks, [18, 19] gene regulatory networks, [20] amino acid interaction networks [21] and protein structural analysis [22, 23] as well. In this work we introduce an asymmetric measure of the directed influence of one amino acid over another amino acid from the same or another protein, use network representation for visualizing it, and illustrate the method with examples.

## Methods

**Sequence selection and alignment:** All the sequence data other than for HIV-1 was obtained from Pfam database. [24] We used the full alignment provided by Pfam. For HIV-1 proteins, the data was obtained from Los Alamos server (https://www.hiv.lanl.gov/). Both the databases give aligned sequences. So separate sequence alignment was not performed. But the alignment was truncated to the reference protein sequence and all sequences having more than 20% gaps were eliminated from the alignment.

**Master sequence:** A master sequence is constructed for the MSA by using the most occurring amino acid in each position. Following the master sequence creation, each amino acid in the MSA is converted into a binary representation, denoting it by “1” if the amino acid at a given position in a sequence is the same (conserved) as the one at the same position in the master sequence, “0” otherwise. Gap is treated as 21^st^ amino acid. When gap becomes the mostly occurring amino acid, the second highest amino acid at that position is taken. While this binary classification may seem restrictive, generalizing this definition did not practically change the conclusions, as discussed later.

**Directed Network:** For any given pair of amino acids (*i*, *j*) two conditional probabilities are calculated:

**a.** *P*(*j* = 1|*i* = 1) = (No. of sequences with *i* = 1 and *j* = 1)/(No. of sequences with *i* = 1 and *j* = 0 or 1), and **b.** *P*(*j* = 0|*i* = 0) = (No. of sequences with *i* = 0 and *j* = 0)/(No. of sequences with *i* = 0 and *j* = 0 or 1)

As a probability *P*(*j* = 1|*i* = 1) and *P*(*j* = 0|*i* = 0) are positive numbers between 0 and 1, We consider an amino acid to be of a certain impact if both *P*(*j* = 1|*i* = 1) and *P*(*j* = 0|*i* = 0) are simultaneously greater than or equal to a value 0 ≤ *γ* ≤ 1 which is suitably chosen depending upon the specifics of the protein and the data set used.

**Statistical analysis:** Statistical significance of the relation between each pair of amino acids was evaluated by a permutation test (2000 random shuffling of the columns). If *p*-value obtained from this statistical test was below 0.01 directional dependence was considered significant and used for further analysis.

**Directed networks:** If there is a directional dependence between two amino acids, treated as nodes in the network terminology, they are considered to be connected by a directed edge. The network representations for these data sets were created by displaying the directed connections.

**Impact Factor:** Impact factor of an amino acid *i* with a cut-off *γ* is defined as the number of amino acids *j* for which *P*(*j* = 1|*i* = 1) ≥ *γ* as well as *P*(*j* = 0|*i* = 0) ≥ *γ*. Impact factor of amino acids is also interesting when considering inter-protein interactions. In this case as well, a similar protocol is followed. MSA of the first protein is joined with the MSA of the second protein, after matching the identities of each of the sequences and ensuring that both the proteins are from the same sample. The rest of the analysis is the same, finding the impact of residue *i* from the first protein, considering residue *j* from the second protein. The impact factor of amino acid *i* on the second protein is defined as the total number of all such residues *j*.

**Dependency Factor:** Similar to the impact factor, we define a dependency factor. The dependency factor of an amino acid *j* is the total number of amino acids which impact it with the same cut-off *γ*.

## Results

### Asymmetric relations and impact calculation

**Fig 1:**
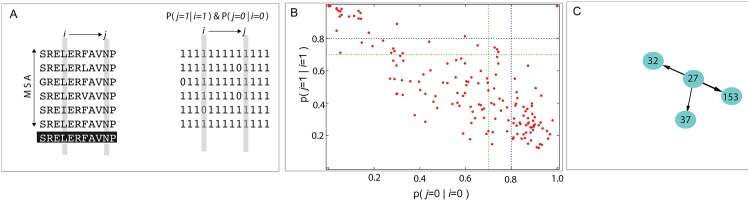
The work flow of creating directed networks. A: Schematic of the Multiple Sequence Alignment and impact calculation B: Example of the impact analysis of one of the amino acids of DHFR performed on 2303 sequences obtained from Pfam database [24] (Pfam Id: PF00186) using PDB id 3QL3 as a reference. The green and blue lines drawn at 0.7 and 0.8 represent the two cut-offs. Amino acid 27 impacts no amino acids with *γ* = 0.8 and 3 at *γ* = 0.7. The data point at (1,1) is the identity relation showing the dependence of 27 on itself. It is not used in the analyses. C: Partial network that was constructed for the impact of amino acid 27 and *γ* = 0.7.

In this work, we introduce directional co-evolutionary interactions among pairs of amino acids, either from the same protein or from two different proteins. We use multiple sequence alignments from homologous proteins to construct a master sequence, relative to which an amino acid in a sequence is coded “1” if it is the most occurring amino acid and “0” if it mutates to other alternatives (see Methods section). The dependence between amino acids *i* and *j*, schematically shown in Fig 1A is evaluated as follows. A definition of asymmetric dependence between amino acids at site *i* and *j* was designed using two conditional probabilities *P*(*j* = 1|*i* = 1) and *P*(*j* = 0|*i* = 0). The first of the two conditional probabilities reflects how amino acid *i*, when it is conserved, imposes conservation on *j* and the second reflects how a mutation in *i* imposes a compensatory mutation on *j*. When both these probabilities are greater than a predefined cut-off *γ*, *i* is considered to have an impact on *j*. The total number of such influences exerted by the amino acid *i* is defined as its impact factor. While there may be alternative creative ways to define asymmetry, the definition used here captures the directional correlations in a simple and intuitive way. The choice of *γ* is discussed later.

**Fig 2:**
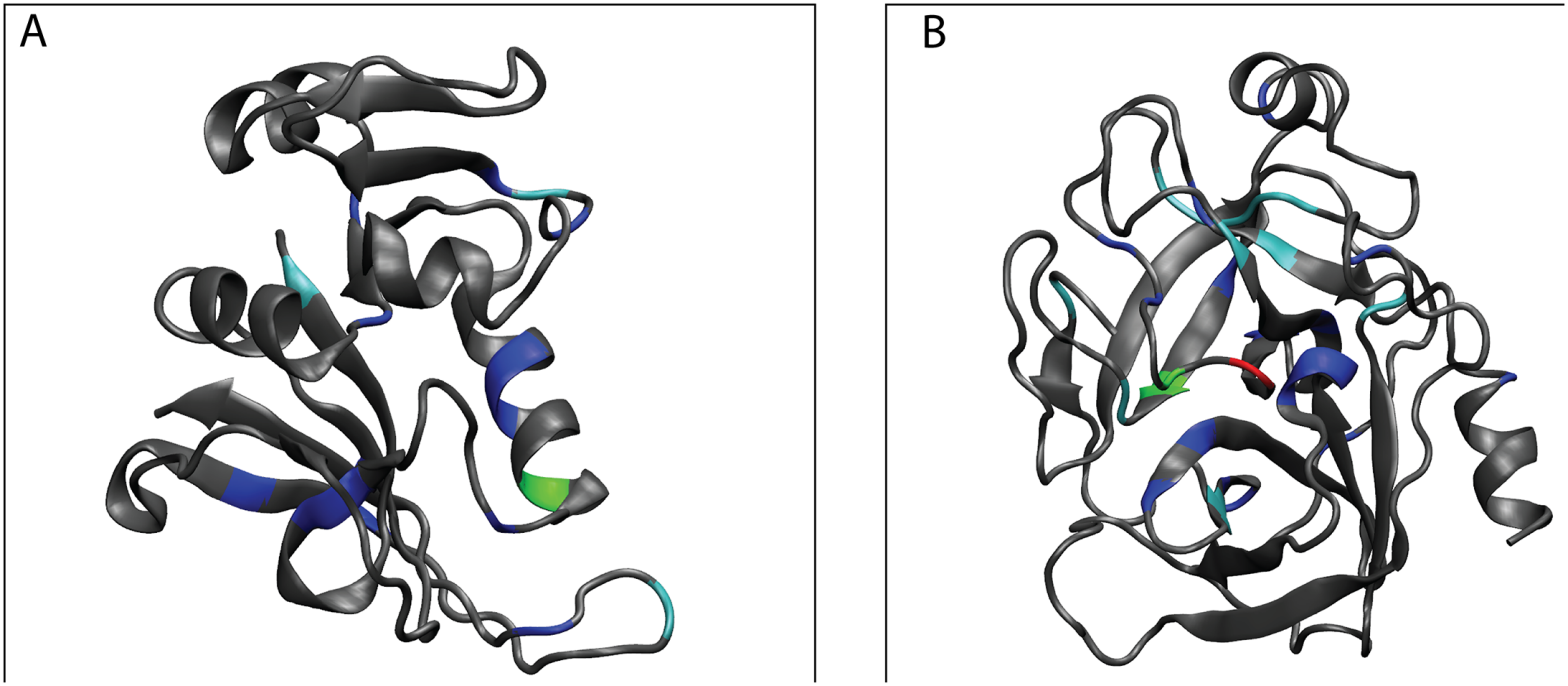
Amino acid residues with non-zero impact factor represented on the three dimensional structures of proteins. A: DHFR. B: serine protease. **Impact factor** (amino acids) for DHFR is: **3** (27), **2** (3, 57, 146), **1** (13, 14, 22, 31, 32, 55, 58, 90, 95, 135, 138, 149) and for serine protease is **8** (196), **3** (140, 194), **2** (19, 34, 102, 142, 182, 183, 184, 216, 228), **1** (29, 32, 40, 42, 57, 58, 100, 122, 136, 168, 189, 191, 201, 211, 226, 237). The coloring convention for PDBs is: Impact 0—gray, Impact 1—blue, Impact 2—cyan, Impact 3—green, Impact 8—red.

We illustrate the calculation of intra-protein impact using two proteins: Dihydrofolate reductase (DHFR) and Serine protease. DHFR plays an important role in the hydride transfer from NADPH to dihydrofolate in the reduction reaction of dihydrofolate to tetrahydrofolate. Fig 1B shows the two conditional probabilities discussed above for all 158 amino acids with amino acid D27 as the reference. It can be seen that on using a cut-off *γ* = 0.8, amino acid 27 does not have an impact on any other amino acid, while with a cut-off of 0.7, it has an impact on three other amino acids L32, D37 and F153. A partial network which shows all amino acids that are impacted by amino acid 27 is then constructed (Fig 1C). Another example of impact calculation was performed on serine protease, an enzyme catalyzing peptide bond cleavage. In the present work, the cut-off was used strictly, without including the few data points that may be slightly less than the cut-off. The impact factor analysis with *γ* = 0.7 identified 16 amino acids from DHFR and 28 amino acids from serine protease and shown on their respective three dimensional structures (Fig 2). The structural mapping shows that the high impact residues could be spread out everywhere, with no specific spatial preference.

### Cut-off and impact factor

To check the sensitivity of the analysis to cut-off parameter as well as to data curation, we repeated the analyses on serine protease. Firstly the analysis was performed by changing *γ* = 0.7 to 0.8. Many residues that were having impact with *γ* = 0.7, continued to appear with *γ* = 0.8 (**Table 1** in S1 File). However, for every amino acid that appeared at *γ* = 0.7, *γ* = 0.8 reduced the number of amino acids it impacted. Thus, while the relative rank order of importance according to either of the choices of *γ* seems to be similar, we further explored if there is a limit to the choice of *γ*. In the network science terminology, the impact factor we defined is one of the centrality measures called the out-degree, which is the number of connections going outward from a given node. [25] It is obvious that as the cut-off is reduced, qualitatively number of nodes as well as the number of outward going connections increase. We make a statistical comparison at the complete network level by using node-degree distribution, [25] which plots number *n* vs. the number of nodes with *n* outward going connections. The node-degree distributions were analysed with different choices of *γ* for serine protease and DHFR (**Figs 1** and **2** in S1 File). The node degree distribution with power-law and poissonian distributions are used to differentiate between ordered and random nature of network connections. [25] In **Figs 1** and **2** of S1 File, one can see that below a certain cut-off the graphs transition from power-law behavior towards poissonian distribution, suggesting a transition to random-networks. The choice of cut-off can thus be limited by these node-degree distributions to avoid the system-level random connections.

When the number of sequences were halved, the master sequence itself can change in principle, especially if a site has a conservation less than 30% or where there are two residues with comparable frequency of occurrence. Among all the proteins we studied, even though there were a few changes in the master sequences when the data set was randomly halved, there were no changes in the amino acid interaction networks, except in the case of Phosphoglycerate kinase (PGK). For PGK one residue position which had appeared in the network had many connections and were not retained when the number of sequences were changed.

### Directed networks and functional relevance

**Fig 3:**
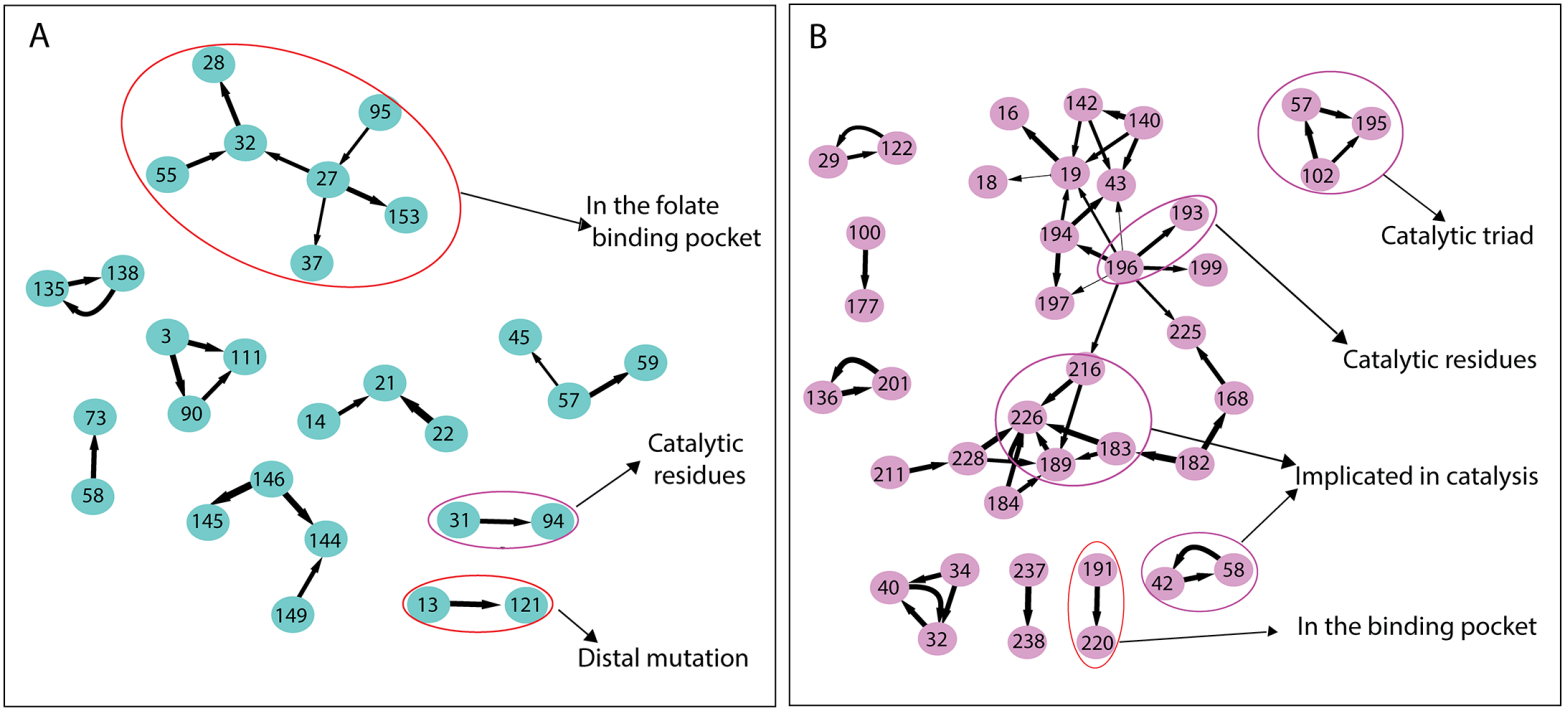
Directed networks and their functional relevance. Residue networks for A: DHFR (PDB Id:3QL3) and B: Serine protease (PDB Id:3TGI). The direction of the arrow shows is in the direction of impact. The thickness of the arrows is proportional to 1/*r* where *r* is the distance between pair of amino acids in the crystal structure. The functional annotation of the amino acids inferred from literature is shown as well.

**Distal mutation in DHFR**: Using the present approach we summarize the compensatory mutations seen in the 2303 DHFR homologous sequences from the Pfam database (Pfam Id: PF00186). Performing the impact factor analysis in DHFR shows that 16 amino acids have impact with *γ* = 0.7. 21 connections were identified using the conditional probability criteria described in the **Methods** section and all of them except one were found to have *p*-value less than 0.01. The residues obtained with *γ* = 0.7 are shown on the three dimensional structure of DHFR labeled with the color coding corresponding to impact factor (Fig 2A). All the identified directed interactions are shown in Fig 3A as a network representation. The residues near to the folate binding pocket are found to have impact on each other. Also the catalytic residue F31 has an impact on catalytic residue I94. More interestingly the mutation at the residue position V13 has an impact on residue G121 which may be essential for maintaining the correlated dynamics between Met20 loop and the region near G121 and hence the catalytic activity. [26, 27] Also it is notable that most of the interacting pair of residues identified in this way are near in structure even though are far in sequence. The residues belonging to each of the disconnected components of the network have comparable conservation.

**Catalytic residues in Serine Protease**: Impact analysis on 14659 sequences obtained from Pfam (Pfam Id:PF00089) homologous to the 223 residue long serine protease shows that there are 28 residues with non-zero impact factor at *γ* = 0.7, 11 with *γ* = 0.8 and 3 with *γ* = 0.9. Amino acids G216, G226, D189 and V183 which were functionally associated with the rates of catalysis experimentally and in the sector analysis (red sector) are captured with this impact analysis. [5] In the case of serine protease also most of the residue pairs identified are near in structure as clear from the network representation of the interactions (Fig 3B). Most interestingly the catalytic triad (H57, D102 and S195) are found to have impact on each other. Also the co-evolving disulphide bond pair C42 and C58 plays important role in catalysis by optimally positioning H57 of the catalytic triad. [28]

**Compensatory mutations in HIV protease and Gag:** HIV protease cleaves the Gag and Gag-Pol polyproteins into individual proteins and hence is vital for the viral maturation. Many of the drugs for HIV target protease. The impact factor analysis on 2550 HIV-1 subtype B protease sequences downloaded from the Los Alamos HIV database (http://www.hiv.lanl.gov/) identified 28 compensatory mutation pairs with *γ* = 0.8. Residue L76 which is located near to the active site cavity is found to have a high impact factor of 6. The compensatory mutation pair V32-M46 has previously been observed experimentally [29] which showed that the reduced replication capacity of the virus due to the mutation V32I is restored by mutation M46I.

HIV virus gains resistance against the protease inhibitors on accumulation of multiple mutations not only in protease but also in the Gag polyprotein [30–32]. Our Gag-Pol inter-protein impact factor analysis with *γ* = 0.9 captured some of the possible compensatory mutations: positions near to the cleavage sites in Gag—A431, G381, P133 acting as compensatory mutations for the protease mutations at L76, L38 and G52 respectively. Changing the cut-off from *γ* = 0.9 to 0.8 resulted in the intra-protein connections increasing from 901 to 1336 and comparably, the inter-protein connections increasing from 266 to 521, highlighting the number of inter-protein compensatory effects.

**Compensatory Mutation in PGK:** Phosphoglycerate kinase (PGK) is involved in the ATP generating step of glycolytic pathway: the reversible reaction of 1,3-bisphosphoglycerate and ADP to 3-phosphoglycerate and ATP. The catalytic residues of PGK is highly conserved across different species. But it is observed that the residue 219 of PGK which is crucial in the dynamics facilitating catalysis is lysine in all Eukaryotes and Bacteria where as it is threonine or serine in *Archaea*. [33] The loss of catalytic activity due to this mutation (K219S) is found to have been restored by compensatory mutations at the positions 239 and 403. [33] Through our impact factor analysis of the Pfam family PF00162 with *γ* = 0.8 we could capture the compensatory mutation at the site 403.

## Discussion

### Directed co-evolution

The present work develops two principles: directed co-evolutionary relationships between amino acids and a quantification of it by counting the number of such dependencies. Amino acids in the primary chain of the protein contribute to its structural stability or function and mutations of these amino acids are differentially tolerated. At a simplistic level, considering the tolerance to the variations in amino acids and/or its neighbors, they may be grouped as: (i) absolutely essential and hence can not mutated, (ii) essential but may tolerate certain substitutions, (iii) essential and tolerate substitutions with suitable compensatory mutations and (iv) not-essential. The present method for identifying directed co-evolutionary relation is mainly to address the amino acids in group (iii). Groups (i) and (ii) are mostly captured by conservation analysis. In fact, if an amino acid is so essential that it was never replaced or evolved among the sequences studied, it will not appear in any co-evolutionary analysis. Further, the directed co-evolution relation developed should not be construed as a description of causal relationships. It represents a statistical summary of the interdependencies among different amino acids while studying large sets of sequence data to identify possible compensatory effects.

In general, when the conditional probabilities of the mutation of one amino acid relative to all other amino acids are studied, such as in Fig 1, the number of relations are few. When the positions *i* and *j* are uncorrelated, *P*(*j* = 1|*i* = 1) = *P*(*j* = 1). Similarly, *P*(*j* = 0|*i* = 0) = *P*(*j* = 0), which is identically 1 − *P*(*j* = 1). So, all the amino acid mutations that are uncorrelated scatter in the anti-diagonal way, as seen in Fig 1B. With a relatively high cut-off *γ*, only a few amino acid relations appear in the zone of interest, which is on the top-right corner. This could be seen from the average number of impact relations that were identified after seeking a significance level *p* < 0.01 (**Table 2** in S1 File). Although we look for directed relations between amino acids, at times the relations may be reciprocated. These reciprocal relations which are signature of co-evolution are incidental, but the focus of the present analysis remains to be the relation between a specific pair, one specific direction at a time.

### Relation to conservation and dependency

**Fig 4:**
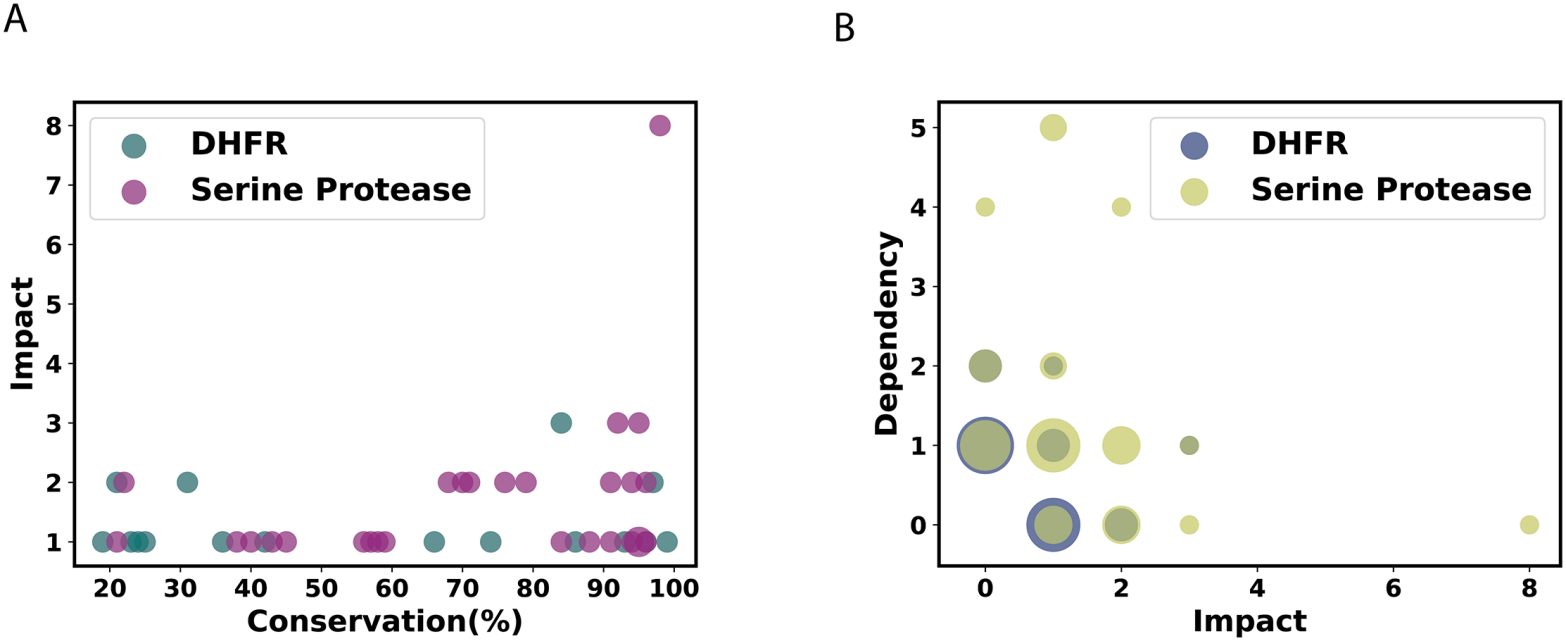
Comparisons of impact with other measures. A: Impact vs. conservation shows that the impact does not trivially repeat the same information contained in conservation. B: Impact vs. dependency shows again in addition to the expected negative correlation between the two, there are several deviations from it. Impact was calculated with *γ* = 0.7. (Size of the marker shows the density of points at that position.)

Functional residues tend to have a higher conservation. Recent studies suggest that most of the information that is contained in the important amino acids identified using SCA is reflected by their conservation. [34] However, under certain conditions, a mutation at these positions can be compensated by changes in other amino acids. We studied the relation of the amino acid impact factors obtained in our calculations to their respective conservation scores. For DHFR and serine protease (Fig 4A) as well as for HIV-1 protease and reverse transcriptase (**Fig 3** in S1 File), we see that the impact factor can not be directly inferred from conservation data alone, and as such it is not a trivial repetition of conservation. Amino acids with low conservation can have a high impact and vice-versa. The spread in conservation for the high impact residues is much broader for DHFR and serine protease as they are obtained from across the species (Fig 4A), compared to that in HIV-1 protease and reverse transcriptase which are obtained from the polymorphisms in the cohort (**Fig 3** in S1 File). Despite these characteristic differences expected in the conservation patterns in these viral and non-viral proteins, the conclusion about lack of its correlation with impact could be seen in both cases.

Fig 4B shows impact versus dependency for DHFR and serine protease. There are several amino acids which have both high impact and dependency. This counter-intuitive behavior comes from some reciprocal relations. It is also possible that these amino acids are intermediates in the interaction network. However for many of these amino acids the higher the impact, the lesser the dependency, which highlights the importance of looking at directed compensatory effects as well rather than co-evolutionary measures alone.

### Three-state model

One apparent limitation that arises from the above analyses is that they use a binary-model: at any position the amino acid corresponds to the one in master sequence or not. Practically, in the data sets we used, we saw a few discrete scenarios where two dominant polymorphisms occurred with comparable frequencies. Hence, without complicating for the theoretical possibility of large number of polymorphisms, we performed a three-state model as the next step towards generalizing our model. In this model we considered residues which occur with a frequency more than 35% at a position to be distinct states. Since there can be at most two states which have a frequency more than 35%, the amino acid code in a sequence is replaced by “1” or “2” depending on the polymorphic state and “0” if it did not belong to either. So the conditional probabilities to be satisfied for position *i* to have an impact on position *j* is: *P*(*j* = 1|*i* = 1) ≥ *γ*,*P*(*j* = 2|*i* = 2) ≥ *γ* and *P*(*j* = 0|*i* = 0) ≥ *γ* or *P*(*j* = 2|*i* = 1) ≥ *γ*,*P*(*j* = 1|*i* = 2) ≥ *γ* and *P*(*j* = 0|*i* = 0) ≥ *γ*

When we repeated the analysis with *γ* = 0.7, the new three-state definition was relevant only to a few amino acids: 1, 6 and 14 amino acids from serine protease, DHFR and PGK respectively. However despite this three state generalisation, these positions from serine protease and DHFR did not appear in the directed co-evolution network. But in the case of PGK, 2 out of 14 had appeared in the network when the binary model is used and the connections involving these residues do not appear when this new definition is used. Thus in the spirit of the inclusive definition of identifying important residues, a more restrictive binary-state definition with slightly more network connections seems suited for the analysis.

### Significance of impact analysis

Using the directed network analysis, several critical amino acids with functional significance could be identified as discussed in the previous section. The perfectly conserved amino acids that never evolve, which are very likely with high functional significance, do not appear in the analysis by the nature of the definition. Those amino acids could anyway be identified using the conservation analysis. The significance of the present analysis should thus be seen as one that identifies amino acids which are likely to have functional repercussions unless compensated, and are not obvious from the standard conservation analyses. Thus the present analysis is to be treated as an inclusive analysis, rather than a comprehensive one, to suggest which amino acids should be included into further analyses—experimental or theoretical. In that sense, the residues requiring the most number of compensatory mutations, may be considered as the significant ones in the analysis. This knowledge may be useful while studying intra- or inter-protein amino acid correlations among large sets of evolutionary or cohort data, and for forming a rational basis for performing site directed mutagenesis experiments. The meaning of the numeric value of the impact factor itself may not be obvious, especially when comparing analyses across two different protein families. However, within one protein family the impact factor rank-orders the different amino acids by summarising the evolutionary data and prioritising them for mutagenesis experiments.

### Resistance models

The notion and definition of directed networks can be generalised to other cases. For example, in analysing the clinical data of the bacterial strains from the group of patients who respond (sensitive) to a drug versus those that do not (resistant), the same principles may be used. Resistance to antibiotics poses a severe public health problem, and usually there is a strong correlation between drug usage patterns in a cohort or a geographic region [35] and the development of bacterial resistance. Mutations of amino acids from critical bacterial or viral proteins that are the targets in drug design, may lead to a fitness advantage. However, these mutations may have to be compensated by other mutations in other sites in the same or other proteins. [36, 37]. For example, in ribosomal protein S12, which is a usual drug target, K42N mutation may be compensated by as many as 35 mutations from both the same protein as well as from others [36]. It is important to identify these compensations that go on in the drug-resistant cohort from the perspective of avoiding problems with secondary drug resistance.

With such background about cohorts and compensatory mutations, one might design questions such as—which are the amino acids *j* that had a compensatory mutation (*j* = 0) in the resistant group when a drug targeting amino acid *i* is used. These mutations in *j* contribute to a structural or functional compensation for a mutation in *i* that made it drug resistant, rather than requiring a reversion of the mutation in *i*. [36] Thus comparing the resistant and sensitive cohorts one can evaluate if the conditional probabilities *P*_*resistant*_(*j* = 0|*i* = 0) and *P*_*sensitive*_(*j* = 1|*i* = 1) exceed a threshold, *γ*. The method is equally applicable when *i* and *j* are from the same protein or from two proteins whose sequences are juxtaposed to perform similar analysis. This analysis is a simpler alternative to the Bayesian analyses that are sometimes used for the specific mutations in the drug-resistance group. [14]

Directed co-evolutionary relationships can be useful either from the protein design or drug design perspective. Considering the compensatory effects, one may plan to add simultaneous mutations along with mutations that contribute to the specific functional gain or design combination therapies such that the primary group of amino acids targeted by the drug, as well as those that undergo consequent mutations are simultaneously targeted.

## Conclusion

We introduced a way to measure and visualise the directed influence of amino acids on one another. The directed influence network summarizes the compensatory mutations under functional constraints in response to changes of key amino acids in homologous sequences. We demonstrate the utility of the method using evolutionary sequences from a few proteins. The principal results seem to be unaffected by changes in parameters and identify effects from compensation to distal mutations, as well as the binding pocket and catalytic residues. The simple and intuitive definition of the directional impact of amino acid interactions can bring a new perspective to the field that had so far relied on symmetric co-evolution.

## Supporting information

S1 File S1 File contains **Table 1**, Variation of impact factor with data set sizes and cut-offs, **Table 2**, Total number of connections identified with a chosen cut-off and the number of connections discarded because *p*-value >0.01, **Fig 1**, The change in node-outdegree distribution with cut-off for serine protease, **Fig 2**, The change in node-outdegree distribution with cut-off for DHFR and **Fig 3**, Impact-conservation analysis for HIV-1 protease and reverse transcriptase.
(PDF)

S2 File Zip file containing the python script and the input files for the analyses discussed in the paper. (ZIP)
