# Supplementary material for: Amino acid impact factor
Source: PLoS One. 2018 Jun 13;13(6):e0198645. doi: 10.1371/journal.pone.0198645 (PMC5999116; doi:10.1371/journal.pone.0198645)
Supplement: S1 File — contains Table 1, Variation of impact factor with data set sizes and cut-offs, Table 2, Total number of connections identified with a chosen cut-off and the number of connections discarded because p-value >0.01, Fig 1, The change in node-outdegree distribution with cut-off for serine protease, Fig 2, The change in node-outdegree distribution with cut-off for DHFR and Fig 3, Impact-conservation analysis for HIV-1 protease and reverse transcriptase. (PDF) [file pone.0198645.s001.pdf]

Supplementary Information

Amino Acid Impact Factor

C. K. Sruthi[1] and Meher Prakash[1]*

[1] *Theoretical Sciences Unit, Jawaharlal Nehru Centre for Advanced Scientific Research*

Bangalore-560064, India

May 27, 2018

Residue Id	Impact factor<br>Full data, $\gamma = 0.7$	Impact factor<br>Full data, $\gamma = 0.8$	Impact factor<br>Half data, $\gamma = 0.8$
19	2	-	-
29	1	1	1
32	1	1	1
34	2	-	-
40	1	1	1
42	1	-	-
57	1	1	1
58	1	1	1
100	1	-	-
102	2	1	1
122	1	-	-
136	1	1	1
140	3	-	-
142	2	-	-
168	1	-	-
182	2	-	1
183	2	-	-
184	2	-	-
189	1	1	1
191	1	-	-
194	3	-	-
196	8	1	-
201	1	1	1
211	1	-	-
216	2	-	-
226	1	1	1
228	2	-	-
237	1	-	-

Table 1. Table showing the variation of impact factor with different data set sizes and with cut-offs 0.7 and 0.8 in the case of **serine protease**

Protein	No. of residues included in the analysis	Reference sequence id	γ	Total no. of connections identified	No. of connections discarded because p-value > 0.01
Serine Protease	216	TRY2_RAT	0.7	48	0
DHFR	158	DYR_ECOLI	0.7	21	1
PGK	398	PGK1_HUMAN	0.8	361	0
HIV protease	99	K03455	0.8	44	16
HIV reverse transcriptase	440	K03455	0.8	340	182
GAG-POL polyprotein	1503	K03455	0.9	2486	1319

Table 2. Table showing the number connections that are identified with a chosen γ as well as the number of connections with p-value > 0.01 that were discarded from the analysis. The Pfam alignment did not have all the residues in the reference PDB, so the analyses do not include all the sequence positions which are present in the pdb.

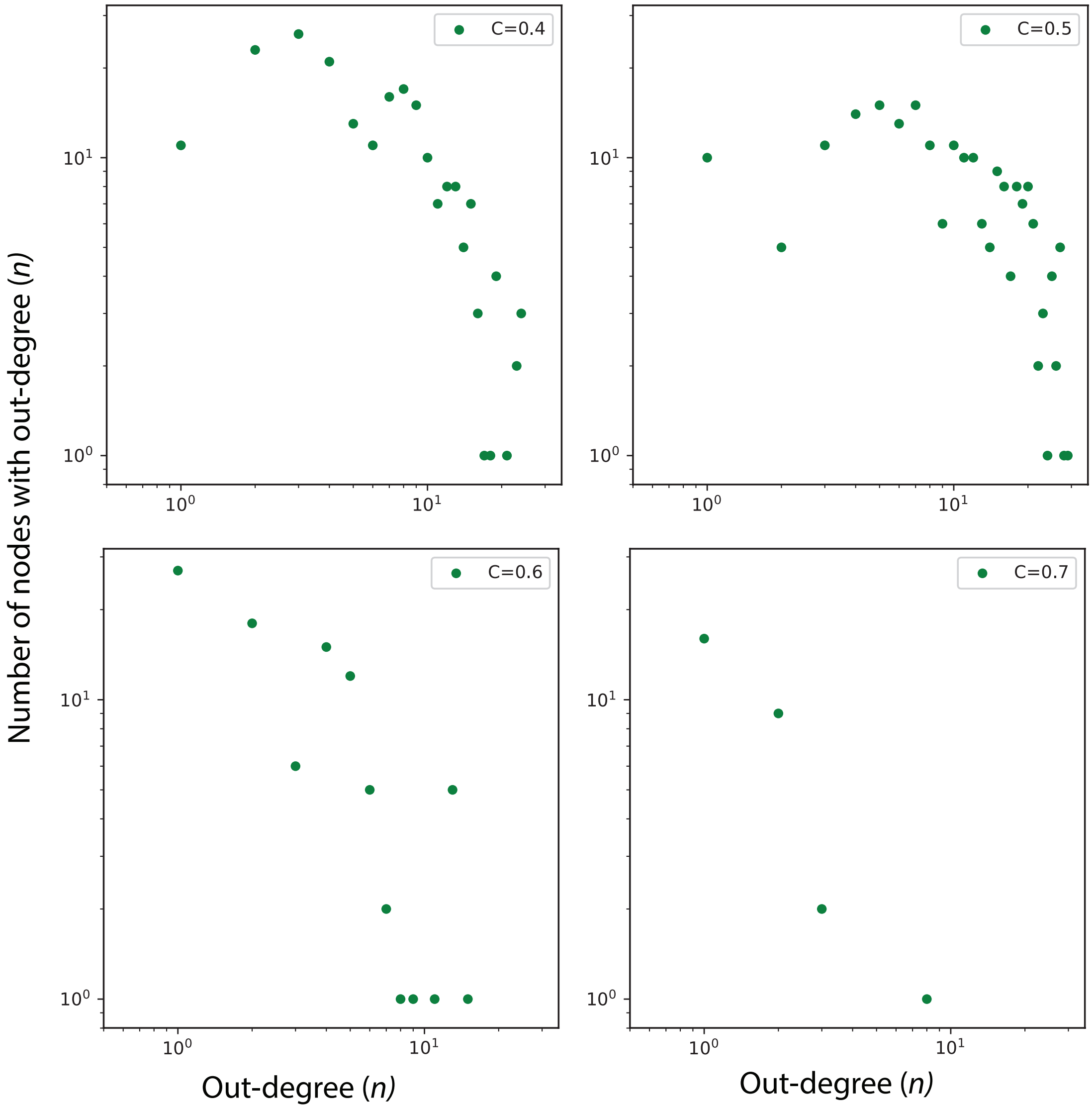

Fig 1. The change in node-outdegree distribution for serine protease. The x-axis indicates the number of out-going connections from a node, and the y-axis shows how many such connections are present. The different subplots represent the same analysis performed with different choices of the cut-off. It can be seen that when the cut-off goes below 0.6, the network begins show a transition from an scale-free to random network.

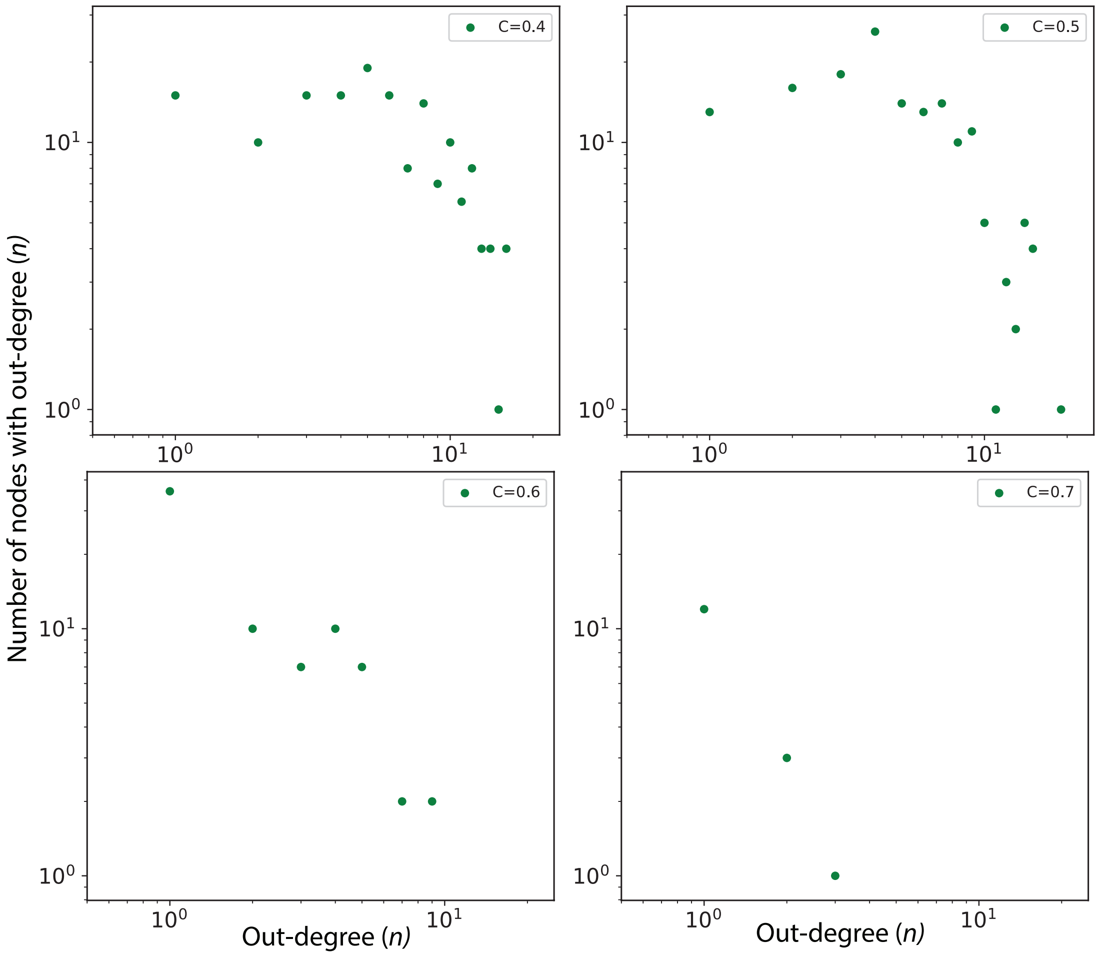

Fig 2. The change in node-outdegree distribution for DHFR. The x-axis indicates the number of outgoing connections from a node, and the y-axis shows how many such connections are present. It can be seen that when the cut-off goes below 0.6, the network begins show a transition from an scale-free to random network.

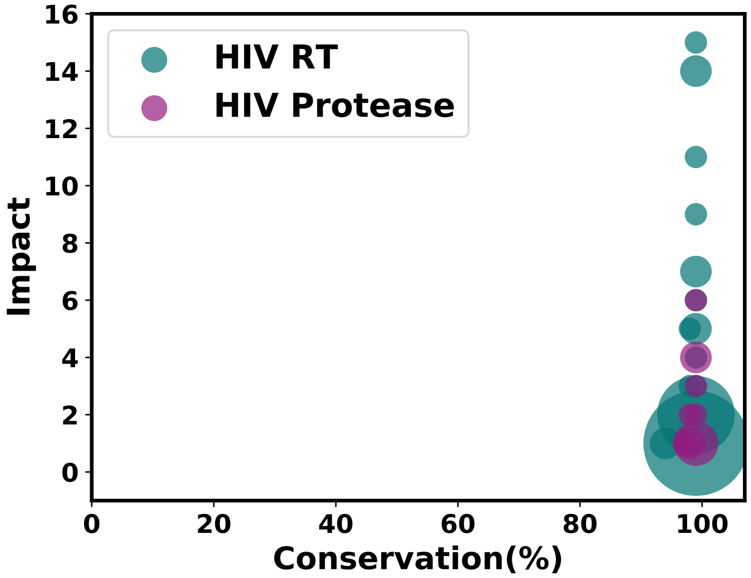

Fig 3. Impact-Conservation analysis showing the residues with impact at $\gamma = 0.8$ on y-axis and conservation on x-axis for HIV-1 protease and reverse transcriptase.
